# Correction: The intellectual disability gene Kirrel3 regulates target-specific mossy fiber synapse development in the hippocampus

**DOI:** 10.7554/eLife.18706

**Published:** 2016-06-16

**Authors:** E Anne Martin, Shruti Muralidhar, Zhirong Wang, Diégo Cordero Cervantes, Raunak Basu, Matthew R Taylor, Jennifer Hunter, Tyler Cutforth, Scott A Wilke, Anirvan Ghosh, Megan E Williams

Martin EA, Muralidhar S, Wang Z, Cervantes DC, Basu R, Taylor MR, Hunter J, Cutforth T, Wilke SA, Ghosh A, Williams ME. 2015. The intellectual disability gene Kirrel3 regulates target-specific mossy fiber synapse development in the hippocampus. *eLife* **4**:e09395. doi: 10.7554/eLife.09395.

Published November 17, 2015

In the material and methods section titled “CHO cell aggregation assay”, we inadvertently omitted 5.5mM glucose from the stated formulation for HEPES Mg^2+^ free (HMF) buffer.

The original sentence “48 hr later, cells were washed with HEPES Mg^2+^ free (HMF) buffer (137 mM NaCl, 5.4 mM KCl, 1 mM CaCl_2_, 0.34 mM Na_2_HPO_4_, 10 mM HEPES, pH 7.4 adjusted with NaOH) and detached from the dishes using 0.01% trypsin in HMF.” should be replaced with the corrected sentence “48 hr later, cells were washed with HEPES Mg^2+^ free (HMF) buffer (137 mM NaCl, 5.4 mM KCl, 1 mM CaCl_2_, 0.34 mM Na_2_HPO_4_, 5.5 mM glucose, 10 mM HEPES, pH 7.4 adjusted with NaOH) and detached from the dishes using 0.01% trypsin in HMF.”

All aggregation experiments were done with HMF media containing glucose and there are no changes to the results.

The article has been corrected accordingly.

